# Effect of Processing Parameters on the Printability and Mechano-Biological Properties of Polycaprolactone–Bioactive Glass Composites for 3D-Printed Scaffold Fabrication

**DOI:** 10.3390/polym17111554

**Published:** 2025-06-03

**Authors:** José I. Contreras Raggio, Miguel Pardo, Pablo Núñez, Carola Millán, Gilberto Siqueira, Humberto Palza, Juan F. Vivanco, Ameet K. Aiyangar

**Affiliations:** 1 Facultad de Ingeniería y Ciencias, Universidad Adolfo Ibáñez, Padre Hurtado 750, Viña del Mar 2520000, Chile; jcontrerasr@alumnos.uai.cl (J.I.C.R.); mipardo@alumnos.uai.cl (M.P.); pabnunez@alumnos.uai.cl (P.N.); 2 Laboratories for Mechanical Systems Engineering, Swiss Federal Laboratories for Materials Science and Technology (EMPA), Überlandstrasse 129, 8600 Dübendorf, Switzerland; 3 Facultad de Artes Liberales, Universidad Adolfo Ibáñez, Padre Hurtado 750, Viña del Mar 2520000, Chile; carola.millan@uai.cl; 4 Laboratory for Cellulose & Wood Materials, EMPA, Swiss Federal Laboratories for Material Science and Technology, Überlandstrasse 129, 8600 Dübendorf, Switzerland; gilberto.siqueira@empa.ch; 5 Departamento de Ingeniería Química y Biotecnología, Facultad de Ciencias Físicas y Matemáticas, Universidad de Chile, Beauchef 851, Santiago 8370456, Chile; hpalza@ing.uchile.cl; 6 Faculty of Medicine, University of Bern, Rosenbühlgasse 27, 3010 Bern, Switzerland

**Keywords:** direct ink writing, additive manufacturing, extrusion-based 3D printing, composite bio-scaffolds, composite ink characterization, bioactive glass, bone scaffolds, PCL

## Abstract

Direct ink writing (DIW) is an attractive, extrusion-based, additive manufacturing method for fabricating scaffold structures with controlled porosity using custom composite inks. Polycaprolactone–bioactive glass (PCL-BG) inks have gained attention for bone applications, but optimizing the formulation and fabrication of PCL-BG-based inks for improved printability and desired mechano-biological properties remains a challenge. This study employs a two-step design to systematically evaluate the effect of three factors in terms of PCL-BG composite printability and mechano-biological properties: ink preparation (acetone or dichloromethane (DCM) as the solvent, and mechanical compounding), the extrusion temperature (90 °C, 110 °C, and 130 °C), and the BG content (0%, 10%, and 20% BG). Pure PCL was used as the control. Rheological, calorimetric, and thermo-gravimetric analyses were conducted before printing. Cylindrical scaffolds and solid wells were printed to evaluate the printability, mechanical properties, and cytocompatibility. The scaffold porosity and pore size were carefully examined. Mechanical tests demonstrated that composite formulations with added BG and higher printing temperatures increased the elastic modulus and yield strength. However, PCL-DCM-BG combinations exhibited increased brittleness with higher BG content. Despite concerns about the toxic solvent DCM, the cytocompatibility was comparable to pure PCL for all ink preparation methods. The results suggest that the interaction between the ink preparation solvent, the BG content, and the printing temperature is critical for material design and fabrication planning in bone tissue engineering applications, providing insights into optimizing PCL-BG composite ink formulations for 3D printing in bone tissue engineering.

## 1. Introduction

Extrusion-based additive manufacturing (AM) has rapidly become a transformative technique in the field of bone tissue engineering, offering the ability to print complex scaffolds from a variety of materials [1,2,3]. One particularly promising approach is direct ink writing (DIW), which enables precise control over the deposition of material through a nozzle using pneumatic pressure, temperature, and extrusion speed [4,5]. This technique allows for the fabrication of scaffolds with intricate geometries that can closely mimic the structure of natural bone, which is crucial for ensuring proper cell infiltration and tissue regeneration. The combination of these materials with DIW’s versatility makes it a strong candidate for personalized, patient-specific bone scaffolds.

Among the wide range of materials used in DIW-based bone scaffolds, polycaprolactone (PCL) has emerged as one of the most widely studied polymers due to its semi-crystalline, biodegradable, and cytocompatible properties. With a relatively low melting point (59–64 °C), PCL allows for easy extrusion and has been shown to support the growth and proliferation of bone cells, making it an ideal material for bone tissue engineering [6,7,8]. However, while PCL offers several advantages, its mechanical properties may not be sufficient for the long-term demands of bone regeneration. To overcome this limitation, bioactive glasses (BGs), particularly Bioglass 45S5^®^, are commonly incorporated into PCL scaffolds. Bioglass is well known for its bone-bonding, osteoinductive, and osteoconductive properties, which can enhance both the mechanical and biological properties of PCL-based scaffolds [9,10,11]. By integrating BG with PCL, scaffolds can exhibit improved bioactivity and mechanical strength, offering a more favorable environment for bone growth and healing.

The preparation of PCL-BG composite inks for DIW, however, presents significant challenges. Traditional melt-mixing methods, where PCL and BG are combined at high temperatures (up to 150 °C), often lead to heterogeneous mixtures. This lack of uniformity in the distribution of BG particles within the PCL matrix can negatively impact the printability and mechanical integrity of the scaffolds, as well as the overall reproducibility of the printing process [12,13,14]. To address this, solvent-based mixing techniques—using solvents such as chloroform, dichloromethane, or acetone—have been explored as alternatives. These methods offer improved homogeneity by facilitating better dispersion of BG particles within the polymer matrix. Solvent-based mixing also enables a reduction in the extrusion temperature, which can help preserve the structural properties of both the PCL and BG components. However, the presence of residual solvents in the final scaffold presents another concern, as these solvents can potentially compromise the mechanical properties and cytocompatibility of the printed structures [15,16]. Acetone is considered a viable solvent due to its lower toxicity profile compared to alternatives such as chloroform or dichloromethane (DCM), thereby reducing the potential for adverse effects in biomedical applications [17].

Previous research has demonstrated the feasibility of fabricating finite-sized (greater than 1500 mm^3^) scaffold structures with precise geometries using PCL-based composite inks through direct ink writing (DIW) [18]. Despite these advancements, several critical questions remain regarding the interplay of key factors involved in the fabrication of PCL-BG scaffolds via DIW. Specifically, the effects of solvent type, extrusion temperature, and BG content on the printability, mechanical properties, and biological performance of the scaffolds have not been fully explored. These factors are integral to the overall success of the fabrication process, as each may interact in complex ways that affect the outcome. For example, the solvent type could influence the viscosity of the ink and its ability to extrude consistently, while the extrusion temperature may affect the fidelity of the printed structures and the dispersion of BG within the polymer matrix. Similarly, varying the BG content could alter the mechanical properties of the scaffold and influence its interaction with cells.

Previous studies [18,19] have highlighted the critical influence of solvent choice, the extrusion temperature, and mixing methods on the mechanical properties and microstructure of 3D-printed polymer-based scaffolds. However, these studies primarily focused on single variables without exploring their interactive effects in a systematic manner. The current study addresses this gap by evaluating the combined impact of solvent type, temperature, and BG content.

The objective of the current work was to systematically investigate how the solvent type, ink extrusion temperature, and BG content influence the printability, mechanical performance, and biological responses of PCL-BG scaffolds fabricated using DIW. By evaluating these factors in isolation and in combination, we aimed to provide a comprehensive understanding of their effects on the final scaffold properties. Specifically, this study focused on the following three aspects: (1) Solvent Type: The solvent used in the preparation of the composite ink can impact the homogeneity of the material and its ability to extrude cleanly. We explored how different solvents, such as dichloromethane and acetone, affect the ink’s viscosity, printability, and microstructure. (2) Ink Extrusion Temperature: The extrusion temperature plays a critical role in determining the printability and mechanical properties of the scaffold. This work examined how varying extrusion temperatures affect the structural fidelity, mechanical strength, and porosity of the printed scaffolds. (3) Bioglass (BG) Content: The concentration of Bioglass in the PCL matrix was varied to assess its influence on the mechanical performance and biological behavior of the scaffolds. We aimed to determine the optimal BG content that provides enhanced mechanical strength without compromising the scaffold’s printability or biological function.

By investigating the primary and interactive effects of these three factors, we sought to identify optimal processing conditions for producing PCL-BG scaffolds that exhibit superior printability, mechanical properties, and biological compatibility. The insights gained from this study will contribute to the development of more reliable and reproducible scaffold fabrication techniques, enabling the production of advanced scaffolds for bone tissue engineering. Ultimately, the findings from this work will provide valuable guidelines for optimizing DIW-based fabrication methods to create scaffolds that meet the structural and biological requirements necessary for successful bone regeneration.

## 2. Materials and Methods

### 2.1. Material Compound

Commercially obtained PCL (Mn 45′000; Sigma Aldrich, St. Louis, MO, USA) was used as the base material for the scaffold. PCL was mixed with a magnetic stirrer with either one of two solvents—acetone (PCL-Ace) or dichloromethane (PCL-DCM)—at a temperature of <60 °C (melting point) and a proportion of 1:2 (PCL:Solvent). A homogenous solution was acquired by mixing for 30 min and applying a temperature just below the boiling point of each solvent. Excess solvent was later evaporated under vacuum for 24 h at room temperature. Furthermore, a third method (PCL-Mini) was processed using a Thermo Scientific HAAKE MiniLab 2 (Dreieich, Germany) at 60 °C, 30 min, and 60 RPM for 7 g of the material. PCL-Ace, PCL-DCM, and PCL-Mini were compared with each other along with unmodified PCL (PCL) as the control in terms of thermal, mechanical, and biological characterization.

Following the characterization of the PCL prepared with different compounding techniques, the two compounding methods with the best performance were used to prepare the polycaprolactone–Bioglass (PCL-BG) composite ink. A custom-produced quaternary bioactive glass (BG: 46.1 mol% SiO_2_, 24.4 mol% Na_2_O, 26.9 mol% CaO, and 2.6 mol% P_2_O_5_ [20]) was used as the composite additive. The Bioglass (BG) was synthesized using a sol–gel process following the methodology detailed in the Supplementary Material and previously reported by [21]. Next, the material was wet-milled in a planetary mill (Retsch PM 400, Haan, Germany) at 300 rpm for 10 min. Zirconia balls of diameters of 1 mm and 3 mm were used as the grinding media with acetone as the dispersing fluid, reaching a mean participle size of 5 µm measured in a LS 13320 laser diffraction particle size analyzer (Beckman Coulter, Brea, CA, USA). The milled suspension was later mixed with PCL following the compounding methods for PCL-ACE and PCL-DCM, as described before. A PCL-BG ratio of 10% and 20% (weight percentage) was defined for the blends of PCL-ACE-BG and PCL-DCM-BG.

### 2.2. Thermal Properties

The crystallinity, glass transition, and melting temperature of PCL and how these properties are affected by the different compounding methods were determined by differential scanning calorimetry (DSC). A heating cycle with temperatures ranging from −90 °C to +100 °C at 20 °C/min was applied to determine the variation of thermal properties prior to and after printing at different temperatures. The first heating curve was reported. The relative crystallinity of the PCL content of the mix was estimated using Equation (1) [22].

(1) $$x_{c}(\%)=100*∆H_{m}/w*∆H_{m}^{0}$$

where $∆H_{m}$ is the heat of fusion of the sample, w is the weight fraction of the polymer on the composite, and $∆H_{m}^{0}$ is the heat of fusion of 100% crystalline pure PCL (135 J/g) [22].

Additionally, thermogravimetric analysis (TGA) was performed on the inks from 0 to 600 °C at 20 °C/min in order to determine the material composition and the variation produced by the compounding methods.

### 2.3. Ink Rheological Characterization

The ink rheological properties were assessed by measurements carried out in an Anton Paar MCR 301 rheometer (Graz, Austria) with a parallel plate configuration with a diameter of 25 mm and a 0.5 mm gap between the plates.

To determine the feasibility of printing the compounds within an appropriate speed range, a viscosity–temperature profile was obtained by a rotational test. Equation (2) was used to determine the theoretical printing speed (where the speed of travel of the nozzle is equal to the exit speed of the material) with respect to the printing pressure, temperature, and nozzle size. A unique pressure level and nozzle were used for the three temperatures (90, 110, and 130 °C) chosen for the experimental setup. Using Equation (3), their estimated printing speeds ranged approximately between 1 and 4 mm/s.

(2) $$v(P,\beta ,\eta )=P*\beta *\eta^{-1}$$

(3) $$v=561.5385*\eta^{-1}$$

where v is the printing speed as a function of the extrusion pressure (P), the nozzle configuration (β), and the viscosity (η) of the sample at a given temperature.

In order to identify whether the printed speed should remain constant along the process, the shear thinning ranges were investigated by means of a rotational test. Furthermore, an oscillatory test was carried out at a fixed frequency of 1 Hz to determine the viscoelastic ink storage (G′) and loss (G″) moduli, and the loss factor (tan δ = G″/G′). G′ and G″ determine the behavior of the printed strut, specifically, if the material will continue to flow after leaving the printer nozzle. For both tests, a minimum of five repetitions were used to ensure the reproducibility of the results. Additionally, to characterize variations in G′ and G″ with change in temperature (e.g., after the ink exits the nozzle), an oscillatory test was carried out from +90 °C to +20 °C. The condition where the loss factor equals one (G′ = G″) [23] represents the ideal balance between the ability of the ink to “flow” out of the nozzle and also to retain its dimensions after exiting without continuing to flow, i.e., “solidification”.

### 2.4. Scaffold Fabrication

A 3D-Bioplotter (EnvisionTec, Gladbeck, Germany) was used for printing the scaffold structures. Syringes loaded with the specific material were mounted in the printer and extruded under pressure at 7.3 bar through a nozzle of a 400 µm inner diameter. Table 1 summarizes the experimental design. Printing was conducted at speeds of 1.2, 2.5, and 3.5 mm/s with temperatures of 90, 110, and 130 °C, respectively. A setup of two fans was placed pointing to the base of the printing sample to cool the printed struts.

The selection of extrusion temperatures (90 °C, 110 °C, and 130 °C) was based on preliminary printability studies and values from the literature [18,24,25] that established these ranges as effective for PCL-based systems. We selected a mid-range temperature (110 °C) based on optimal print fidelity, with lower and higher temperatures providing boundaries for comparative analysis.

The samples were designed in the 3D-Bioplotter design software (Magics, EnvisionTec, Germany) with a 50% porosity, a 400 µm pore size, a 400 µm targeted strut size, and a 320 µm layer height, printed with an orthogonal pattern infill and continuous strand in a cylindrical geometry of diameter (Ø) = 10.5 mm and height (H) = 17.36 mm. Six cylinders were printed for each subgroup to be used for mechanical characterization, according to ASTM D695-15 [26].

### 2.5. Scaffold Microarchitectural Properties

#### 2.5.1. Porosity

The overall porosity of the scaffolds was determined based on Archimedes’ principle by means of the fluid displacement method using a fluid with known density—in this case, ethanol (ρ_ethanol_) [27]. A digital balance (Mettler Toledo AT400, Columbus, OH, USA) was used to record the weight of each dry sample, and with a buoyant basket, the weight under liquid to obtain the experimental porosity using Equation (4).

(4) $$Porosity=1-(P-T)/{(V}_{total}*\rho_{ethanol})$$

where P is the dry weight, T is the weight submerged in the liquid, $V_{total}$ is the overall volume of the sample, and ρ_ethanol_ is the density of the liquid.

The external dimensions of the samples were measured with calibrated digital calipers (±0.02 mm), with the diameter measured at three different locations on the specimen (the first, middle, and last layers). Furthermore, at least eight measurements were taken for the specimen height with the axial rotation of the sample to ensure a representative diameter and height dimension. The mean values represent each specimen’s diameter and height.

#### 2.5.2. Pore Size

In addition to estimating the overall porosity and to have a better understanding of the printing consistency with the inner structure of each printed sample, layer-by-layer measurements of the pore size and strut thickness were carried out to ensure that the designed microarchitecture was reproduced with high fidelity throughout the printed scaffold.

A camera attached to the Bioplotter recorded an image after each layer was printed. A custom Python (version 3.9) script was developed to identify the printed struts and to calculate the orthogonal inter-strut distance, which provided the corresponding cross-sectional pore size. A region of interest (ROI) was manually selected within the first layer. This ROI was automatically applied to each successively printed layer. A total of 1470 pores per specimen were measured to obtain the mean pore size as well as the distribution across the printed layers. Furthermore, the pore area, the perimeter, and the circularity of the pores were analyzed. Equation (5) was used for circularity calculation.

(5) $$Circularity=4\times \pi \times \frac{Pore\;area}{{Pore\;perimeter\;}^{2}}$$

The acquired information was further used to predict the overall porosity of the specimens; in short, both a mathematical model and a machine learning algorithm were implemented to correlate the pore size value and the overall porosity of the sample. This prediction was compared with the experimental value of Section 2.5.1.

### 2.6. Scaffold Mechanical Properties

Printed scaffold specimens were tested in compression in a universal testing machine (Zwickroell, Ulm, Germany) with a 10kN load cell. The specimens were placed in metal endcaps to reduce end-effects [18]. Testing was carried out under a displacement control protocol with a crosshead displacement rate of 1.00 mm/min. Specimen deformation was measured with displacement transducers on either side of the specimen. The test was carried out according to ASTM D695-15 [26].

The apparent elastic modulus (E_app_) was calculated by linear regression of the nominal stress–strain data from the linear portion of the curve, generally between 0 and 2% strain. The corresponding yield stress and strain were acquired using the offset method, displacing the linear segment by 1% [28] along the X axis. The Y and X coordinate values of the intersecting point on the nominal stress–strain curve were used to determine the offset yield stress and strain, respectively.

### 2.7. Scaffold Biological Assessment

To assess the cytocompatibility of the PCL samples (without BG) prepared with the different compounding methods, solid wells (diameter = 5 mm, height = 2 mm) were printed at a constant temperature of 110 °C. The samples were sterilized in 70% ethanol for 30 min, and then rinsed with PBS. Human bone progenitor cells (HBPCs) were isolated from bone marrow samples, obtained with informed consent and local ethical approval (EKOS 22/193). Samples from three different patients were used. Cell seeding was performed by adding 200 µL of the HBPC suspension (20,000 cells/cm^2^) onto the samples for incubation at 37 °C with 5% CO_2_ and 100% humidity. The samples were kept in standard cell culture conditions for up to 7 days, and the culture medium was replaced on day three.

To assess cell attachment and spreading, the cells were stained after 24 and 72 h of culture to assess their actin cytoskeleton (Alexa Fluor 488-labelled phalloidin (1:200) A12379, Invitrogen, Waltham, MA, USA) and nuclei (4′,6-diamidino-2-phenylindole (DAPI (1:1000), Sigma D9542), before acquiring images with a confocal laser-scanning microscope (LSM780, Zeiss, Oberkochen, Germany). Additionally, cell viability was assessed by quantifying cell metabolic activity on days 1, 3, and 7 with an alamar blue assay (alamarBlue™ Cell Viability Reagent DAL1025, Invitrogen) according to the manufacturer’s instructions. The cells were incubated for 2h at 37 °C in the alamar blue/cell culture mix (10% *v*/*v*) before reading the fluorescence signal intensity at 530 and 635 nm in a spectrophotometer. Six samples were used for each recorded time and experiment.

### 2.8. Statistical Analysis

In order to evaluate the effects of the three factors—the solvent type, the extrusion temperature, and the BG content—on the porous scaffold’s geometrical, mechanical, and biological properties, we implemented a two-step experimental design. In the first step, we assessed the effect of temperature (three levels) and compounding methods (four levels) without any added BG content. In the second step, the two best compounding methods and extrusion temperatures were selected. The third factor—% of BG content—was then included in the analysis.

The results are expressed as group means ($\bar{x}$) with standard deviations (±SD). In all cases, Analysis of Covariance (ANCOVA) and Tukey’s Honest Significant Difference (HSD) post hoc test were performed using R (version 4.2.1). The porosity was the covariate where needed. The significance level was set to α = 0.05 and was reduced by Bonferroni adjustment for multiple testing corrections when needed. The normality was checked using the Shapiro–Wilk test, whereas the equality of variances was checked by Bartlett’s test.

## 3. Results

### 3.1. Thermal Properties

**Figure 1:**
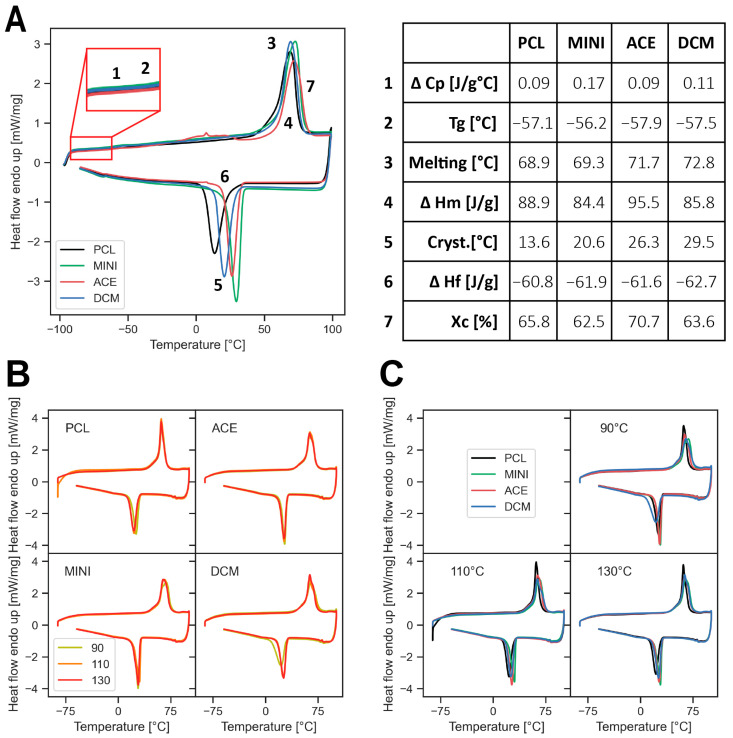
PCL thermal properties. (**A**) DSC of different inks, with numbers indicating thermal properties summarized in the inset table, where Cryst. refers to the crystallization temperature. A magnified region highlights Tg and ∆Cp. (**B**) DSC of printed structure at different temperatures grouped by the input ink and (**C**) grouped by the printed temperature.

DSC experiments aimed to compare the samples before and after printing; in order to determine any physical change which could potentially influence the mechanical properties of the printed scaffold, the first heating curve was reported (Figure 1A). The inks presented variability in their specific heat capacity (Cp), crystallinity (X_c_), melting (T_m_), and crystallization temperature (T_c_) (Figure 1B) based on the mixing technique. Figure 1C presents the same data as in Figure 1B, but is grouped based on the printing temperature. Using acetone as the mixing solvent appeared to result in a slight increase in crystallinity. While variations in the melting temperature (T_m_) were relatively smaller—up to 4 °C—differences in crystallization temperature were larger (up to 16 °C), ranging from 13.6 °C for pure PCL to 29.5 °C for PCL-Mini. The printed temperature and compounding methods are compared in Figure 1B and Figure 1C, respectively.

**Figure 2:**
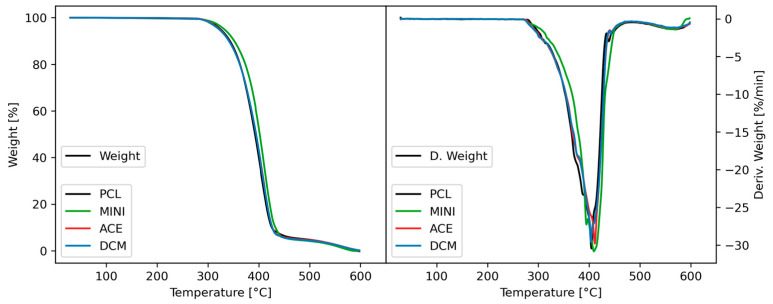
Inks thermal properties. TGA of the inks prior to printing, where the left graph highlights the weight loss and the right graph depicts the derived weight by time.

TGA experiments sought to determine the maximum safe temperature before material degradation sets in. The TGA curves present similar behavior for the different compounding methods (Figure 2). It can be observed that the degradation step ranges between 350 to 435 °C, with a temperature of the maximum decomposition rate of 407 °C. Furthermore, no changes in the weight percentage were observed on the solvent evaporation ranges.

### 3.2. Rheological Properties

**Figure 3:**
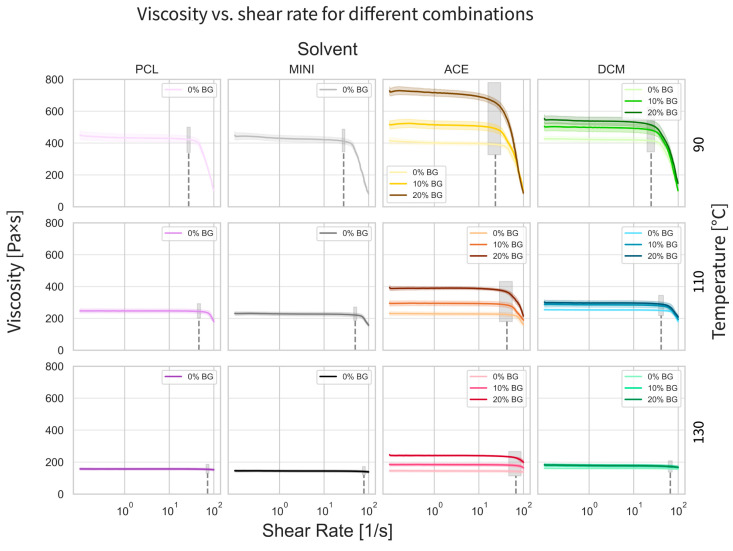
Rheological properties. A steady-shear rotational test for the samples at all combinations. The lines represent the average and blurred area with a 95% confidence interval. The grey areas, if present, determine the printing range for the current configuration, with their average value indicated by the dotted line.

The rheology results show, predictably, a decrease in viscosity with an increase in temperature (90, 110, and 130 °C). Figure 3 further reveals that the shear-thinning behavior occurred at a higher shear rate with this increase in temperature (i.e., the start of the shear-thinning behavior is shifted to the right along the *x*-axis). Figure 3 also reveals the interactive effects of temperature, solvent type (acetone or dichloromethane), and filler concentration (% BG). Larger amounts of BG tend to increase the viscosity of the ink, while the viscosity decreases with increasing temperature. However, the variations in the viscosity are substantially larger for the inks prepared with acetone compared to those prepared with dichloromethane.

The addition of BG results in an increase in rheological properties, in particular viscosity, and in a decrease in the shear rate for the initiation of shear-thinning behavior (Figure 3). This effect is noticeable primarily at lower temperatures (e.g., 90 °C compared to 130 °C).

**Figure 4:**
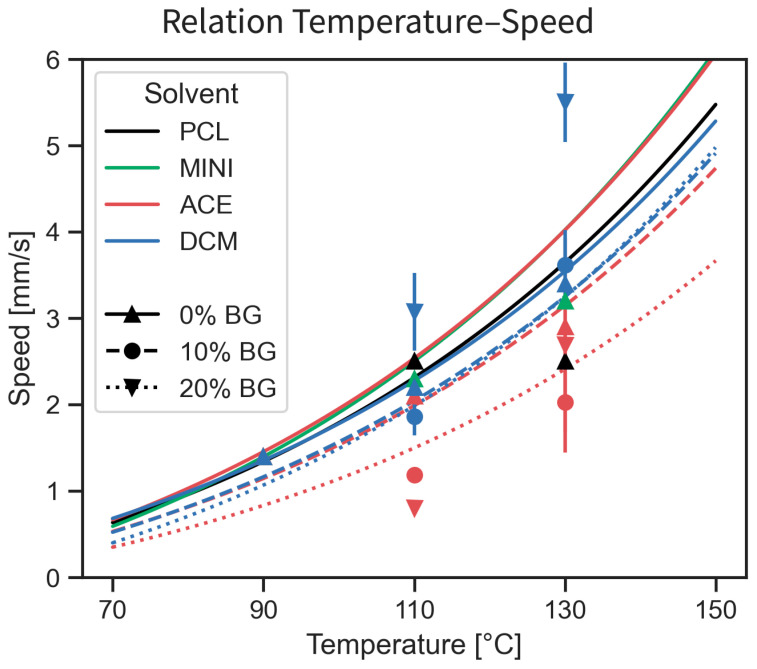
Validation of the printing speed model. The temperature–speed relation for an optimal strut size; markers represent the actual combination used during printing, with vertical bars representing the variation during printing as the SD.

The theoretical printing speed was calculated for the different compounds and temperatures. The temperature sweep reveals only a minor variation for the different compounding methods between the ideal temperature–speed correlation (computed using Equation (3)) and the experimental values used for the experiment. This difference in the theoretical speed for each compound is lower than the sensitivity of the 3D-Bioplotter configuration. Nevertheless, composite inks exhibit a considerably larger difference (greater than the sensitivity of the 3D-Bioplotter) between the experimental and predicted printing speed, as shown in Figure 4.

Oscillatory tests showed a positive loss factor (G″ > G′) for all three temperatures tested. Hence, preserving the printed shape requires an external mechanism to quickly decrease the temperature of the extruded filament to a point where G″ ≤ G′ [18].

### 3.3. Scaffold Microstructural Characterization

**Figure 5:**
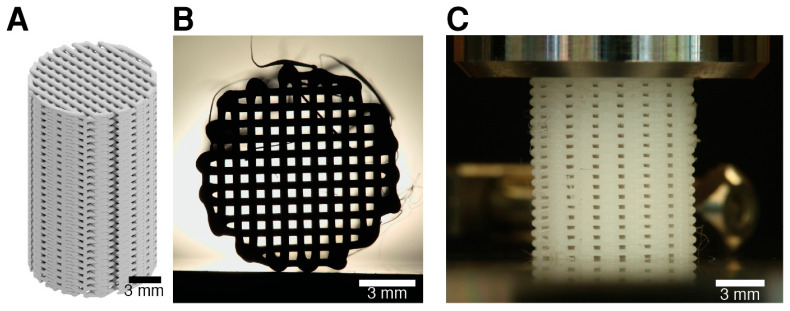
Scaffolds’ microstructural representation. (**A**) CAD design of a scaffold, (**B**) an image of a scaffold under a source of light showing the excellent alignment of the pores though the whole scaffold (H = 17.5 mm; 55 layers), and (**C**) a lateral picture of a scaffold inside endcaps, before being mechanically tested showing a homogenous lateral porous structure.

**Figure 6:**
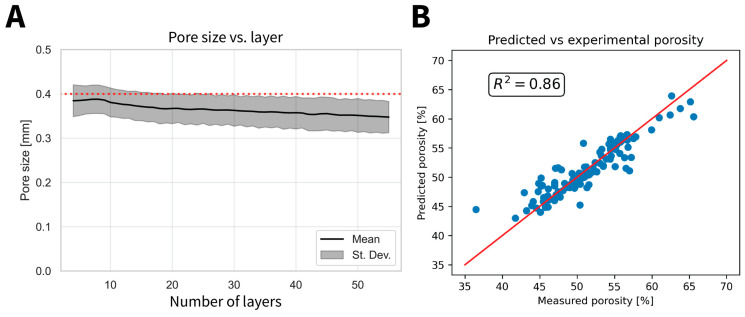
Scaffolds microstructural properties. (**A**) The mean pore size per printed layer; the grey shaded area represents the standard deviation. The dotted red line shows the design pore size of 400 µm. (**B**) A ccomparison of the overall porosity measured by the Archimedes method (measured porosity) with that acquired of the central inner structure with the code (predicted). The line shows the diagonal as a perfect match (slope = 1) and R^2^ is the coefficient of determination.

In general, the structures were printed with consistency, as shown in Figure 5. Nevertheless, detailed layer-by-layer mapping of the inner structure revealed a progressively diminishing pore size with the addition of successive layers (Figure 6A).

Overall the porosity of the specimen could also be estimated from the captured images using the code (R^2^ = 0.86), revealing a linear relationship (Equation (6)) between the pore size and the overall porosity of the specimens measured using the Archimedes method (Figure 6B).

(6) Predicted porosity (%)=190.92×Pore size−17.94

The histogram of the pore size demonstrates the homogeneity of the extrusion and the effect of temperature on the pore size. Supplementary Figure S1A shows that high extrusion temperature (130 °C) exhibits the lowest pore size statistic mode of 360 µm in comparison with the designed value (400 µm) for 0% BG. Moreover, 130 °C shows the lowest average pore size (361 ± 33 µm), followed by 110 °C (374 ± 32 µm) and 90 °C (378 ± 38 µm). On average, 110 °C exhibits the highest overall frequency and closest pore size to the designed value. The circularity is close to the desired circularity (square = ~0.785) (Supplementary Figure S1B). There was no evidence of any significant effects of the different processing parameters on pore circularity. Nevertheless, an increase in circularity occurs in the first 10 layers, after which it remains more or less constant for the rest of the printing.

### 3.4. Scaffold Mechanical Properties

**Figure 7:**
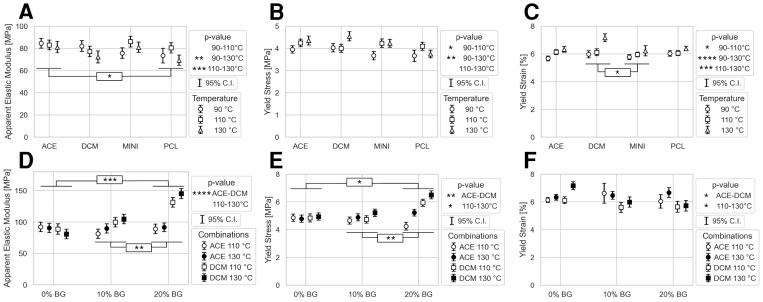
A mechanical analysis of the samples: (**A**) The apparent elastic modulus, (**B**) yield stress, and (**C**) yield strain for 0% BG displayed by the compounding method. (**D**) The apparent elastic modulus, (**E**) yield stress, and (**F**) yield strain for 0, 10, and 20% BG displayed by the BG concentration. Markers represent the mean value, with whiskers representing the 95% confidence interval. Statistics between groups are shown in the graph or legend under, with the *p*-values being *p* < 0.05: *; *p* < 0.01: **; *p* < 0.001: ***; *p* < 0.0001: ****. Note: plotted values for E_app_ and the apparent yield stress are based on ANCOVA, i.e., after correcting for the porosity in the individual specimens.

To test the effect of the compounding method, the printing temperature, and the percentage of the BG on the scaffold mechanical behavior, uniaxial compression testing was performed (Figure 7A–F). The apparent elastic modulus (E_app_), yield stress, and yield strain were reported. Supplementary Figure S2 shows the average stress–strain curves of the mechanical characterization for each condition.

Since calculations are based on nominal rather than the actual (i.e., accounting for porosity) specimen cross-section area, the specimen porosity exhibited a large correlation with E_app_ and yield stress [29]. Hence, an ANCOVA was performed, with porosity as a covariate, to account for the confounding effect of porosity. The yield strain was assessed using a regular ANOVA, since the yield strain was not sensitive to porosity variations [29]. The goal in this study was to assess the mechanical performance across the groups relative to each other, rather than the absolute mechanical properties of the individual scaffold specimens. Hence, we report apparent values (e.g., the apparent elastic modulus) rather than “true” values. Further, we admit that the obtained values would be proportionally scaled to the “actual” or “true” values when applying the effective cross-sectional area as opposed to the nominal cross-sectional area. However, when assessing the relative influence of the main processing parameters (temperature, solvent type, and composite BG content) on mechanical performance, the key confounding factor becomes the variability in the porosity and pore sizes across the printed specimens, rather than the theoretical, or designed value of the pore size/porosity. To address this issue, porosity was added as an additional factor in the statistical evaluation of the relative mechanical performance across groups. However, rather than simply including porosity as an additional fourth factor, it was included as a covariate in the ANCOVA analysis (Analysis of Covariance), which allowed us to focus on identifying and directly correcting any variability attributable to the specimen porosity induced into the mechanical testing results.

The results are shown in Figure 7, and both sets of values (with and without porosity correction) have been included in Supplementary Tables S1 and S2 for completeness, where porosity was standardized to 53.4% and 49.8%, respectively, by applying ANCOVA [30]. In short, ANCOVA operates by fitting a linear model where the dependent variable (e.g., Eapp or yield stress) is regressed on both the independent categorical variables (e.g., solvent type, extrusion temperature, and BG content) and the continuous covariate (porosity). This allows for the partitioning of variance attributed to porosity, ensuring that comparisons between groups reflect differences in mechanical performance independent of porosity fluctuations.

In the first step of the statistical design of the experiment, the effects of temperature and compounding methods were assessed. Mechanical properties were only mildly influenced by the compounding method (Figure 7A–C; Supplementary Tables S1 and S2). Based on ANCOVA, only the following result was statistically significant: E_app_ [PCL-Ace (83 ± 7.1 MPa) > PCL (74.4 ± 8.0 MPa)]. The yield stress [PCL-DCM and PCL-Ace (4.2 ± 0.3 MPa) > PCL (3.8 ± 0.3 MPa)] trended towards significance. Any potential differences in yield strain across the different compounding methods were not statistically different. On the other hand, the extrusion temperature exhibited a stronger influence on the mechanical properties. For example, printing at 130 °C resulted in the lowest E_app_, (Figure 7A) and the highest yield strain (Figure 7C). PCL-DCM at 130 °C exhibited the highest yield stress (Figure 7B) and strain (Figure 7C), indicating a potential interactive effect between the printing temperature and the compounding solvent.

In the second step of the statistical analysis, this study further evaluated the effect of BG content after selecting the two best performing levels from the compounding and printing temperature groups—PCL-Ace and PCL-DCM, and 110 °C and 130 °C—respectively (Figure 7D–F; Table S2). The addition of BG displayed a significant main effect on E_app_ and yield stress, in particular with the addition of 20% BG content (E_app_ [0% BG (87.9 ± 15.8 MPa) ~ 10% BG (93.7 ± 15.3 MPa) < 20% BG (114.1 ± 15.5 MPa)], and yield stress [0% BG (4.9 ± 0.6 MPa) ~ 10% BG (4.9 ± 0.5 MPa) < 20% BG (5.5 ± 0.5 MPa)]). Nevertheless, the effects appeared to be modulated by the solvent used and the printing temperature, as the largest difference was at 20% between solvents (Figure 7D–F). The temperature and compounding solvent also appeared to influence E_app_ and yield stress. Specimens printed at 130 °C exhibited higher values than 110 °C, on average. Similarly, inks prepared with DCM were stiffer and stronger compared to those prepared with ACE, on average. However, the effects were much lower at 10% BG, with the most pronounced effects observed at the highest BG content tested. The combination of 20% BG prepared with DCM and printed at 130 °C displayed the highest apparent stiffness (E_app_) and yield stress, considerably higher than the total average [E_app_: PCL-DCM-20% (145.1 ± 4.2 MPa) > Group average (98.5 ± 28.4 MPa); yield stress: PCL-DCM-20% (6.5 ± 0.1 MPa) > Group average (5.1 ± 1.3 MPa)]. Finally, neither the BG content nor compounding solvent appeared to significantly influence the yield strain, although a trend towards an interactive effect with temperature was visible (Figure 7F).

### 3.5. Scaffold Biological Properties

**Figure 8:**
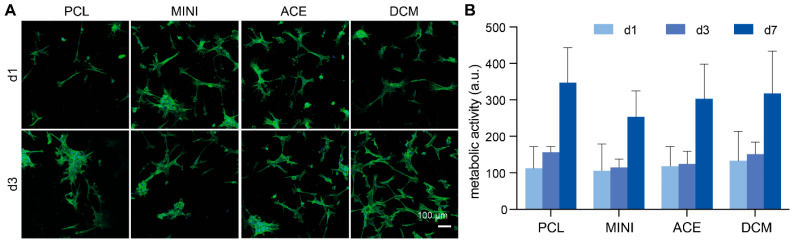
Biological properties. (**A**) HBPCs cultured on samples prepared by the different compounding methods, stained for actin cytoskeleton (green) and nuclei (blue). (**B**) The metabolic activity of HBPCs after 1, 3, and 7 days of culture on samples of the different groups (*n* = 2 for d1, *n* = 3 for days 3 and 7).

In order to study the cytocompatibility of samples prepared by the different compounding methods, the attachment and spreading of HBPCs were assessed after 1 and 3 days of culture (Figure 8A). The cells showed limited cell attachment and spreading at both timepoints, with no apparent difference visible between the different groups. Quantifying the metabolic activity of HBPCs after 1, 3, and 7 days of culture on the samples confirmed these observations and only at the latest timepoint could a marked increase be observed for all groups (Figure 8B). Notably, no statistically significant difference could be observed between the different groups at any time point.

## 4. Discussion

While PCL-BG composites are attractive candidate materials to fabricate scaffolds for bone tissue engineering applications, developing custom formulations and printing precise and reproducible structures of finite sizes (e.g., >1000 mm^3^) with a controlled inner architecture remains challenging. This study evaluated, in a systematic manner, the primary and interactive effects of (1) the kind of processing, (2) the ink extrusion temperature, and (3) the BG content on the printability and mechanical properties of PCL-BG scaffold structures fabricated by DIW. Porous scaffolds were analyzed for their micro-architectural and mechanical characteristics, as well as their cytocompatibility using HBPCs.

Three PCL-BG composites (0, 10, and 20 wt.% BG) were assessed in terms of different process parameters (solvents and extrusion temperature). In the first step, 0% BG was characterized in order to assess the effect of compounding methods on extrusion feasibility, mechanical properties, and cytocompatibility.

Although the results suggested that there was not a significant difference between the compounding methods with respect to scaffold mechanical properties and cytocompatibility, PCL-Mini (mechanical compounding) required the longest processing times, as each series produced a limited amount of 7 g/h. Therefore, the longer process time makes it susceptible to contamination within the mechanical compounding device. Hence, further evaluations of the PCL-BG composite were limited to ink preparation based on solvent mixing with either acetone or dichloromethane. The effect of the printing temperature on the fidelity and the mechanical properties of printed structures was also considered in this first step, showing that printing at the lower temperature of 90 °C led to longer printing times with more failures in the samples’ geometry and size. These negative effects were exacerbated in the initial attempts to print with BG at these temperatures. Hence, the second part of this study involving PCL-BG composites was limited to two preparation methods (PCL-DCM and PCL-ACE) and two printing temperatures (110 °C and 130 °C).

### 4.1. Printability

Rheological experiments become relevant as the key DIW-related settings are dependent on the viscosity and related properties of the ink. The speed at which the ink flows through the nozzle is given by the relation between the pneumatic pressure of the DIW system, the dimensions of the nozzle, and the viscosity of the ink. It is essential to match this speed with the printer speed to have an optimal deposition of the material. Therefore, knowing the viscosity of the samples and how it changes with the different compounding methods and extrusion temperatures becomes fundamental for a controlled printing process, consequently obtaining controlled structures with high geometrical fidelity. Notably, all material–temperature combinations exhibited a zero-shear viscosity plateau at low shear rates, and a pseudo-plastic flow, wherein the fluid viscosity decreases with increasing shear, was manifested at high shear rates. Similar behaviors have been shown for PCL-based composites [31,32]. Since higher viscosity materials require higher extrusion pressures, printing in the shear thinning range potentially leads to an easier and smoother print [33]. Nevertheless, for all the cases evaluated in this study, the printing range was within the zero-shear viscosity plateau, mainly due to characteristics of the 3D-bioplotter, implying a slower printing rate but a more consistent process.

The viscosity–speed relation obtained from Equation 3 for different temperatures provides a rough estimate of the necessary parameters for an optimal print for each of the different combinations. This reduces the time and effort from an otherwise arduous trial-and-error method to determine the 3D printing parameters for new ink development, while simultaneously improving the reproducibility and fidelity of the printed porous structures. Nevertheless, the PCL-BG composite inks exhibited larger deviations between the predicted and the actual printing speed than single materials, suggesting that further work is necessary to refine the equation to provide better estimates for composite formulations.

Understanding the behavior of the ink upon printing is also relevant, as prior knowledge of the relative values of G′ and G’‘ will predict if the printed strut can support its own weight, or if it will continue to flow, thus deviating from the designed strut thickness. The formulations used in the current study displayed behavior where G″ > G’, i.e., the material would continue to flow on deposition, thus compromising architectural fidelity. We have followed the explanation presented in the review of printable bioinks by Schwab et al. [34] relating the post-printing shape retention to ink rheological properties and extended it to our composite polymer ink. Accordingly, the condition of G′ = G″ represents the flow point for the ink/melt, which usually occurs at a higher stress than the yield point, below which shape is retained. Ideally, the ink should display G″ > G′ under “high” deformation/stress conditions (e.g., within the nozzle), thus enabling flow, but switch to G′ > G″ at “low” deformation/stress conditions with minimal delay (e.g., after exiting the nozzle), enabling shape retention. Shape retention and, consequently, print fidelity are better for inks with higher yield points [34]. In our case, since G″ continued to be larger than G′ even at “low” stress, it indicated poor shape retention ability unless additional measures were undertaken. We addressed this issue by adding an external system of forced cooling, which enabled faster cooling of the freshly printed specimen. Moreover, this system appears to be even more necessary based on Figure 1A, where the “solidification temperature” (theoretically equivalent to the case where G″/G′ = 1) is substantially lower than the melting temperature. This difference requires a cooling system to quickly decrease from the extrusion temperature to this low transition temperature to preserve the dimensions of the printed structure. Inadequate consideration of these factors could lead to either poor reproducibility or the low fidelity of the printed samples.

A brief note on scaffold pore sizes: Previous research has shown the critical influence of scaffold pore size on bone tissue engineering outcomes. Across various studies, pore sizes larger than 300 μm consistently promoted key biological responses such as osteoblast adhesion, proliferation, and vascularization—essential for effective bone regeneration. This body of evidence supports the conclusion that optimizing pore dimensions is fundamental in scaffold design to enhance cell ingrowth and new bone formation. Thus, from a bio-application perspective, the mean pore size is an important variable affecting the ability of bone scaffolds to stimulate cell ingrowth and new bone formation [35,36,37]. However, obtaining the correct estimate of the mean pore size and the variability within the fabricated scaffolds is equally important.

**Figure 9:**
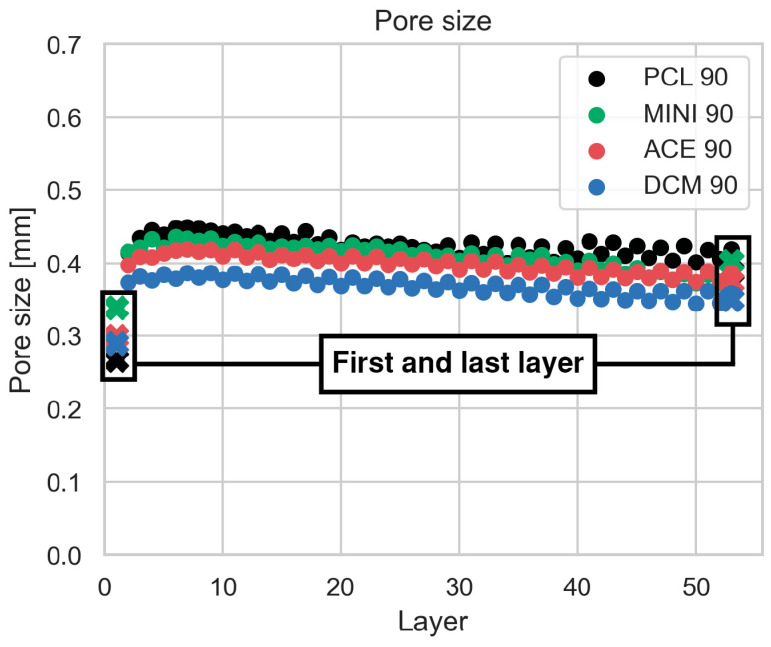
Comparison of pore size measurement methods. The graph shows the average pore size per printed layer, with dots representing the mean values obtained using the method described in this paper. The black region of interest highlights the first and last layers, where crosses indicate pore sizes measured using a conventional method (i.e., acquiring and analyzing only the first and last layer via microscopy). This comparison illustrates the discrepancy between methods across individual layer values.

Generally, only the mean of the outermost upper- and lower-layer pore size values is used to determine the average pore size of the whole structure. However, the pore size of the first printed layer is strongly influenced by the printing bed [38,39], whereas the upper layer is the result of all the accumulated errors of the whole structure, making the measured values less representative of the whole specimen. Figure 9 represents the comparison between both methods for a group of samples and provides an example of the incorrect estimation of pore sizes when assessment is limited to the outer two layers of the scaffold, rather than performing a more comprehensive assessment of the whole scaffold. Layer-by-layer pore size mapping revealed an initial increase in the first few layers, followed by a small but consistent, progressive decrease in pore size. In contrast, simply measuring the pore size from the first and last printed layers would suggest, erroneously, a linearly increasing trend leading to inaccurate conclusions on specimen pore sizes and pore size distribution. While Figure 9 displays results from a randomly selected specimen, our pore size measurement results were broadly consistent across all sample groups from this perspective.

Using the information given by the code [S_R_EL2], the values can be analyzed as part of a normal curve in terms of the pore size and frequency. Supplementary Figure S1A,B displays the effect of extrusion temperature on the mean scaffold pore size: scaffolds printed at higher extrusion temperatures resulted in a smaller pore size in comparison with those printed at lower temperatures. This effect is attributed to the efficiency of the cooling system and the longer time required to cool down the material from 130 °C; therefore, the material spreads more, reducing the pore size. Moreover, frequency determines the distribution of the pore sizes. A low frequency value represents a larger deviation from the ideal, designed pore size in the printing process and thus, a less controlled extrusion. For example, printing at 90 °C resulted in lower frequency values and, hence, larger variability between the compounding methods, which is attributed to the poor extrusion flow on account of high viscosity at the lower temperature. Moreover, the printing speed was at the lower limit (1 mm/min), resulting in longer printing times, which is often accompanied by a higher probability of errors. Similar characteristics were found with 130 °C, where the low frequency can be explained by the high printing speeds producing irregular extrusion, while requiring more time for cooling. Finally, 110 °C resulted in pore sizes closest to the designed pore size. Therefore, 110 °C is recommended in order to optimize the quality and consistency of the scaffold specimens.

The use of solvents reduced the compounding temperature and increased the homogeneity of the mixture. However, different solvents exhibited a variation in thermal and mechanical properties, particularly in the presence of BG as a composite additive.

### 4.2. Thermal, Mechanical, and Biological Properties

To test whether the compounding method, extrusion temperature, or the addition of BG changes the compressive mechanical properties of the scaffolds, quasi-static tests were performed for all conditions. Additionally, nominal mechanical properties, i.e., the mechanical properties—E_app_ and yield stress—computed using nominal specimen cross-sectional area rather than the actual or effective area of porous specimens, are strongly influenced by the variability in porosity between specimens. This is often qualitatively self-evident, but printing porous structures inherently creates variability in pore sizes and, consequently, variability in the effective cross-sectional area of the specimens being tested. To account for this variability, we included porosity as a covariant. The ANCOVA method allowed us to correct for the effect of variations in cross-sectional area, as represented by variations in porosity, for the purpose of statistically assessing the influence of the main factors in question, i.e., the solvent, printing temperature, and BG content.

Although no significant differences could be observed regarding the use of either acetone or dichloromethane as a solvent on the specimen mechanical properties, differences for the composite PCL-BG scaffolds were indeed significant. The effects were most notable with the highest BG content composite ink (20% BG) printed at the highest temperature (130 °C), suggesting a strong interactive effect between the added BG content and temperature when DCM is used as the solvent for ink preparation. While the apparent elastic properties might imply a positive effect—the printed specimens were substantially stiffer and stronger—the post-yield behavior (Supplementary Figure S2) revealed the specimens to be significantly more brittle, with samples shattering on failure, which may not be a favored failure mode. This effect is attributed to a potential interaction between DCM and the milled BG, suggesting an influence of the solvent on the mechanical properties of the printed scaffold, which might already occur at the ink preparation phase, but is likely exacerbated when printing is conducted at higher temperatures. The macro-mechanical behavior suggests either a chemical change or a polymorphic effect occurring in this configuration when composite inks, particularly with higher BG content, are prepared with DCM and then printed at higher temperatures. It must be noted, however, that these differences were not obvious in specimens prepared with DCM, but without BG (Supplementary Table S1). Similar findings were reported by Vallejos et al. [18], where increasing BG content led to reductions in mechanical properties, likely due to particle agglomeration and inefficient stress transfer at the PCL-BG interface. Additionally, the use of dichloromethane (DCM) in the ink formulation may have introduced micro-porosity within the matrix [40], exacerbating brittleness. Initial DSC results revealed small changes in the crystallinity of the PCL specimens: the crystallinity of the PCL-Ace and PCL-DCM specimens was approximately 7% higher and 5% lower than the pure PCL specimens, respectively (Figure 1). Directly comparing the two solvents, the crystallinity of the PCL-DCM specimens was approximately 12% lower than PCL-Ace. Consequently, one should expect a potentially stiffening effect with acetone rather than dichloromethane. While results from the first step (Figure 7A) narrowly support this assumption, the substantial stiffening along with loss of ductility for the configuration with DCM at higher BG content and higher printing temperatures appears counterintuitive, suggesting additional interactions occurring within this specific combination. Further studies are planned to identify the underlying mechanisms to explain this phenomenon.

Using solvents to modify mechanical properties and facilitate the compounding and extrusion process has been a matter of ongoing debate, given that residual solvents may negatively impact biological performance [13]. In our study, we evaluated the effect of solvents on the compounding process and observed that the cytotoxicity, attachment, and metabolic activity of HBPCs were comparable to the control group (PCL) across all experimental groups. This suggests that the solvents used were effectively evaporated from the prepared scaffolds.

Unmodified PCL is generally known to provide limited support for initial cell attachment and spreading—an observation that aligns with our findings. Therefore, composite fabrication strategies are often required to improve cell–material interactions [41]. During the cell adhesion process, both the chemical properties of the biomaterial and the surface topography play essential roles in supporting initial adhesion as well as the subsequent stages of viability and proliferation. In our study, all solvent-treated groups were able to support initial cell adhesion (1 day), demonstrating biocompatibility.

The attachment process and cellular extension behaviors typically seen in HBPCs adhered to scaffolds [42] were evident in our specimens. Actin staining revealed cytoskeletal structures with cytoplasmic extensions, indicative of early and tight adhesion to the material surface. In the future, it would be valuable to include studies using scanning electron microscopy (SEM) to better determine whether cell morphology varies depending on the scaffold composition, and to assess how these variations influence cell attachment, spreading, and the formation of key structures such as filopodia and lamellipodia—both of which are essential for the proper adhesion of cells to biomaterial surfaces [43].

While the addition of 0.4 wt.% bioactive glass (BG) to PCL scaffolds has previously been reported to have no significant effect on early cell attachment and metabolic activity [44], the incorporation of higher BG concentrations, as in our study, may influence initial cell–material interactions and potentially affect downstream osteogenic differentiation. Particularly in the case of DCM-based specimens with higher BG content, additional physico-chemical changes may be introduced that can influence mechanical properties. These effects should be carefully considered when selecting solvents for mixing and composite ink formulation. Future studies should explore the interactive effects of DCM and BG content on biological responses, with a special focus on the osteogenic differentiation of HBPCs.

### 4.3. Limitations

The current work showed how compounding methods affect different properties of printed scaffolds. Additionally, while solvent evaporation was performed at room temperature for 24 h, trace amounts of residual solvents (acetone, DCM) may persist. Although biological assays confirmed cytocompatibility, future work should incorporate vacuum drying or thermal treatments to further minimize solvent residues. Furthermore, there may be a lingering effect on the chemical properties of the biopolymer—PCL, in this case—in terms of the surface texture and chemical bonds. The current study did not investigate these potential effects, which will be considered in future work.

Finally, this study revealed strong interaction between solvents and BG in terms of mechanical characterization, although this was only observed qualitatively. Future work should address this interaction with a more detailed statistical design of the experiment to explicitly quantify the extent of the main and interactive effects of the parameters investigated in this study.

## 5. Conclusions

This study investigated the influence of the solvent type, the ink extrusion temperature, and the amount of composite additive (BG content) on the printability, mechanical properties, and cytocompatibility of PCL-BG scaffolds fabricated using DIW. The results demonstrated not only the significance of the individual process parameters but also the impact of their interactions. Notably, scaffolds prepared with DCM and 20% BG content displayed higher stiffness and strength (yield stress), especially when printed at 130 °C. On the other hand, macro-mechanical observations—e.g., the brittle failure behavior—imply a chemical change or polymorphic effect occurring with this particular combination. Although biological assessments showed no significant (toxic) effects of DCM on cell viability, as, presumably, the solvent was adequately evaporated during preparation, its use is nevertheless discouraged due to known carcinogenic effects.

Second, the inner architecture and printability were influenced by the extrusion temperature and BG concentration. Low printing temperatures (90 °C) substantially increased printing times and extrusion errors, while higher printing temperatures (130 °C) tended to reduce the geometrical fidelity of the printed structure. Thus, an “intermediate” extrusion temperature of 110 °C was ideal for an optimal printing speed and inner quality of the scaffolds.

This study highlights the nuances of the interactive effect of the solvent type, the temperature, and the amount of composite additive on the printability and biomechanical properties of the printed structures. In particular, the interactive effects between PCL-DCM-20% BG at 110 °C and the potential associated polymorphic changes merit further investigation before we can fully endorse an optimal material combination for scaffolds in bone tissue engineering. The interplay of relative advantages and disadvantages of each parameter choice must be quantitatively assessed and considered in order to optimize scaffold performance. Since no solvent type emerged as a “clear winner” in this study, solvent-free methods of melt extrusion should continue to be explored in future research.

## Figures and Tables

**Table 1:** Processing parameters: compounding type, extrusion temperature, and filler concentration (% BG content) for the different inks for direct ink writing.

Compounding		Extrusion Temperature			
Acronym	Solvent	90 °C	110 °C	130 °C	
PCL (control)	None	0%	0%	0%	BG% Content
PCL-Mini	Mechanical compounding	0%	0%	0%	
PCL-DCM	Dichloromethane	0%	0% 10% 20%	0% 10% 20%	
PCL-Ace	Acetone	0%	0% 10% 20%	0% 10% 20%	

## Data Availability

The original contributions presented in this study are included in the article/supplementary material. Further inquiries can be directed to the corresponding authors.

## Supplementary Materials

The following supporting information can be downloaded at https://www.mdpi.com/article/10.3390/polym17111554/s1, Supplementary Figure S1. Pore characterization; Supplementary Figure S2. Average Stress-Strain curves; Supplementary Table S1: Summary of mechanical properties for 0% BG; Supplementary Table S2: Summary of mechanical properties for Ace and DCM at 110 and 130 °C.
