# Supplementary material for: Effect of Processing Parameters on the Printability and Mechano-Biological Properties of Polycaprolactone–Bioactive Glass Composites for 3D-Printed Scaffold Fabrication
Source: Polymers (Basel). 2025 Jun 3;17(11):1554. doi: 10.3390/polym17111554 (PMC12158094; doi:10.3390/polym17111554)
Supplement: Supplementary file 1 [file polymers-17-01554-s001.zip › polymers-3533452-supplementary.pdf]

Supplementary material

Bioglass (BG) Synthesis via Sol–Gel Method

The bioglass (BG) used in this study was synthesized via a sol–gel process, based on the methodology described in Ref. [21]. The precursor materials included tetraethyl orthosilicate (TEOS, Aldrich, 98%), triethylphosphate (TEP, Aldrich, 99.8%), sodium nitrate (Riedel-de Häen, 99.5%), and calcium nitrate tetrahydrate (Merck, 99%).

Hydrolysis of TEOS and TEP was catalyzed using a 0.1 M HNO_3 solution, with a molar ratio of (HNO_3 + H_2O)/(TEOS + TEP) = 8. TEOS was hydrolyzed first, followed by sequential addition of the remaining reagents, spaced by 45-minute intervals under constant stirring. After the final reagent was added, the sol was stirred for an additional 60 minutes.

The resulting sols were transferred to high-density polyethylene vessels and aged for 3 days at room temperature. Gels were then dried at 60 °C for 3 days and 130 °C for 2 days, respectively. Dried gels were ground using an analytical mill and heat-treated at 700 °C in air in a ceramic crucible using an electric furnace. The resulting powders were manually milled in an agate mortar and sieved through a 200-mesh sieve (<75 μm) prior to use.

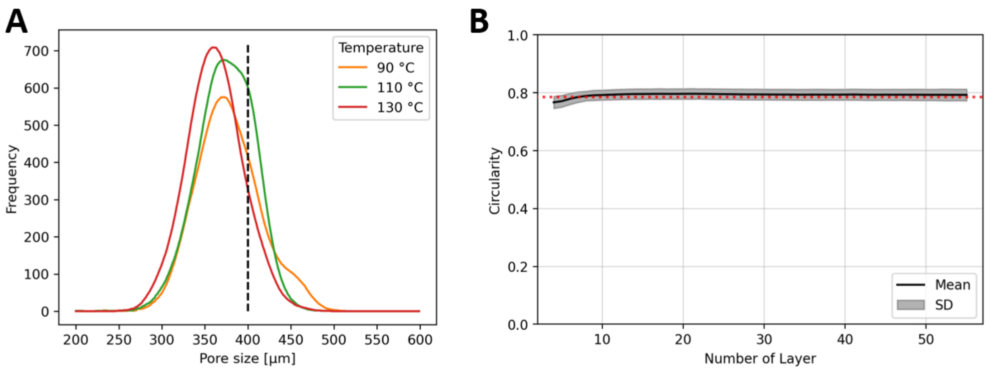

Supplementary Figure S1. Pore characterization. (A) Pore size histogram of pore distribution grouped by extrusion temperature for 0% BG. Black dashed line represent the designed pore size. (B) Circularity changes along the printed layers. Black line represents the cumulative mean for all measured pores and grey region represents the standard deviation. Red dotted line represents the ideal value for a square (~0.785).

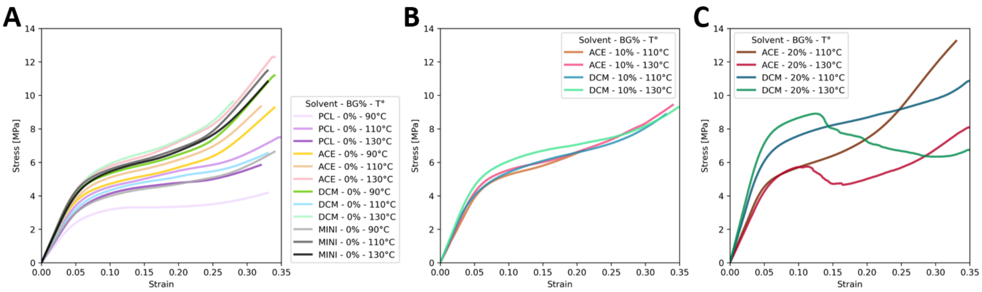

Supplementary Figure S2. Average Stress-Strain curves. Average per combination of conditions of the mechanical behavior of (A) 0% BG, (B) 10 % BG, and (C) 20% BG. Legend shows compounding method (Solvent), percentage of Bioglass (BG%) and extrusion temperature (T°).

Supplementary Table S1: Summary of mechanical properties for 0% BG. Materials and Temperatures are referred as Pooled when grouped. Values are displayed as mean ± standard deviation. ANCOVA was performed – using porosity as a covariant – and standardized values are displayed for a porosity of 53.4%.

Material	Temperature [°C]	Measured Porosity [%]	Measured Pore Size [μm]	E_{app} [MPa]	App. Yield Stress [MPa]	App. Yield Strain [%]	ANCOVA E_{app} [MPa]	ANCOVA App. Yield Stress [MPa]
PCL	90	61.3 ± 4.7	403.6 ± 40.4	52.9 ± 19.3	2.6 ± 0.8	6.0 ± 0.2	73.5 ± 8.3	3.7 ± 0.3
	110	55.6 ± 0.7	390.1 ± 32.6	75.0 ± 4.4	3.8 ± 0.1	6.1 ± 0.2	80.5 ± 5.7	4.1 ± 0.2
	130	55.6 ± 1.2	386.9 ± 33.2	64.1 ± 4.1	3.4 ± 0.2	6.4 ± 0.2	69.3 ± 5.7	3.7 ± 0.2
	**Pooled**	**57.5 ± 3.9**	**393.6 ± 36.4**	**64.0 ± 14.4**	**3.3 ± 0.7**	**6.2 ± 0.3**	**74.4 ± 8.0**	**3.8 ± 0.3**
PCL-DCM	90	50.1 ± 0.6	352.3 ± 24.2	90.7 ± 5.7	4.5 ± 0.2	6.0 ± 0.3	82.2 ± 6.0	4.0 ± 0.2
	110	56.5 ± 0.6	388.5 ± 25.4	69.9 ± 2.3	3.6 ± 0.2	6.1 ± 0.3	77.2 ± 5.9	4.0 ± 0.2
	130	48.4 ± 2.1	345.3 ± 25.3	84.9 ± 5.4	5.2 ± 0.1	7.2 ± 0.4	72.3 ± 6.7	4.5 ± 0.3
	**Pooled**	**51.6 ± 3.8**	**361.7 ± 31.2**	**81.8 ± 10.1**	**4.4 ± 0.7**	**6.4 ± 0.6**	**77.3 ± 7.6**	**4.2 ± 0.3**
PCL-Ace	90	53.7 ± 0.7	370.9 ± 28.3	83.6 ± 3.5	3.9 ± 0.1	5.7 ± 0.2	84.7 ± 5.4	4.0 ± 0.2
	110	53.0 ± 3.2	365.8 ± 31.8	83.1 ± 10.5	4.3 ± 0.6	6.1 ± 0.2	83.1 ± 5.4	4.2 ± 0.2
	130	49.1 ± 1.8	349.6 ± 25.9	92.4 ± 3.6	4.9 ± 0.3	6.3 ± 0.3	81.1 ± 6.4	4.4 ± 0.2
	**Pooled**	**51.9 ± 2.9**	**362.7 ± 30.3**	**86.4 ± 7.7**	**4.4 ± 0.6**	**6.0 ± 0.4**	**83.0 ± 7.1**	**4.2 ± 0.3**
PCL-Mini	90	56.9 ± 1.5	392.5 ± 28.3	67.7 ± 4.9	3.2 ± 0.2	5.8 ± 0.2	75.8 ± 6.1	3.7 ± 0.2
	110	50.0 ± 1.2	353.9 ± 25.4	94.6 ± 4.6	4.7 ± 0.2	6.0 ± 0.2	86.2 ± 6.1	4.2 ± 0.2
	130	50.9 ± 3.2	360.3 ± 29.4	87.8 ± 5.4	4.6 ± 0.5	6.2 ± 0.5	81.1 ± 5.8	4.2 ± 0.2
	**Pooled**	**52.6 ± 3.7**	**362.6 ± 30.5**	**83.4 ± 12.7**	**4.2 ± 0.8**	**6.0 ± 0.4**	**81.0 ± 7.3**	**4.0 ± 0.3**
**Pooled**	90	55.5 ± 4.8	377.4 ± 37.7	73.7 ± 17.9	3.6 ± 0.8	5.9 ± 0.3	79.0 ± 10.5	3.8 ± 0.4
	110	53.8 ± 3.1	374.4 ± 32.8	80.7 ± 11.2	4.1 ± 0.5	6.1 ± 0.2	81.8 ± 9.5	4.1 ± 0.4
	130	51.0 ± 3.5	361.4 ± 33.3	82.3 ± 11.9	4.5 ± 0.8	6.5 ± 0.5	76.0 ± 10.1	4.2 ± 0.4
**Pooled**	**Pooled**	**53.4 ± 4.3**	**370.9 ± 35.2**	**78.9 ± 14.3**	**4.1 ± 0.8**	**6.2 ± 0.4**	**78.9 ± 30.0**	**4.1 ± 1.2**

Note: Mechanical property calculations (E_{app}, σ_{app} and ε_{app}) are based on nominal cross section area. ANCOVA allows to correct for the effect of variations in cross sectional area, as represented by variations in porosity, for purposes of statistically assessing the influence of the main factors in question.

Supplementary Table S2: Summary of mechanical properties for Ace and DCM at 110 and 130 °C. Materials, Temperatures and BG % are referred as Pooled when grouped. Values are displayed as mean ± standard deviation. ANCOVA was performed – using porosity as a covariant – and standardized values are displayed for a porosity of 49.8%.

Material	Temperature [°C]	BG [%]	Measured Porosity [%]	Measured Pore Size [µm]	E_{app} [MPa]	Yield Stress [MPa]	Yield Strain [%]	ANCOVA E_{app} [MPa]	ANCOVA App. Yield Stress [MPa]
PCL-Ace	110	0	53.0 ± 3.2	365.8 ± 31.8	83.1 ± 10.5	4.3 ± 0.6	6.1 ± 0.2	92.0 ± 3.9	4.9 ± 0.1
		10	49.9 ± 8.4	358.3 ± 47.0	81.0 ± 17.0	4.6 ± 1.5	6.6 ± 0.9	81.1 ± 3.8	4.7 ± 0.1
		20	46.1 ± 2.2	337.3 ± 26.6	99.3 ± 13.9	4.9 ± 0.4	6.0 ± 0.6	88.9 ± 4.0	4.2 ± 0.1
		**Pooled**	**49.7 ± 5.8**	**353.1 ± 37.3**	**87.8 ± 15.7**	**4.6 ± 0.9**	**6.3 ± 0.7**	**87.3 ± 11.7**	**4.6 ± 0.4**
	130	0	49.1 ± 1.8	349.6 ± 25.9	92.4 ± 3.6	4.9 ± 0.3	6.3 ± 0.3	90.3 ± 3.8	4.8 ± 0.1
		10	50.1 ± 3.0	354.3 ± 32.6	88.8 ± 12.0	4.8 ± 0.5	6.5 ± 0.3	89.6 ± 3.8	4.9 ± 0.1
		20	50.7 ± 4.5	357.3 ± 30.2	88.9 ± 17.7	5.1 ± 1.2	6.7 ± 0.4	91.4 ± 3.5	5.2 ± 0.1
		**Pooled**	**50.0 ± 3.3**	**354.2 ± 30.3**	**89.9 ± 12.3**	**4.9 ± 0.8**	**6.5 ± 0.4**	**90.4 ± 11.2**	**5.0 ± 0.4**
	**Pooled**	**Pooled**	**49.8 ± 4.6**	**353.7 ± 33.8**	**88.9 ± 13.9**	**4.8 ± 0.9**	**6.4 ± 0.5**	**88.9 ± 22.8**	**4.8 ± 0.8**
PCL-DCM	110	0	56.5 ± 0.6	388.5 ± 25.4	69.9 ± 2.3	3.6 ± 0.2	6.1 ± 0.3	88.6 ± 4.3	4.8 ± 0.2
		10	52.2 ± 6.9	386.9 ± 44.0	93.0 ± 30.1	4.3 ± 1.4	5.6 ± 0.5	99.6 ± 3.9	4.7 ± 0.1
		20	47.1 ± 3.2	344.1 ± 30.4	138.9 ± 11.8	6.5 ± 0.8	5.6 ± 0.5	131.3 ± 3.9	5.9 ± 0.1
		**Pooled**	**51.9 ± 5.7**	**373.4 ± 41.0**	**100.6 ± 34.3**	**4.8 ± 1.5**	**5.8 ± 0.5**	**106.5 ± 12.0**	**5.2 ± 0.4**
	130	0	48.4 ± 2.1	345.3 ± 25.3	84.9 ± 5.4	5.2 ± 0.1	7.2 ± 0.4	80.8 ± 3.8	4.9 ± 0.1
		10	50.6 ± 3.0	357.3 ± 29.8	102.3 ± 18.0	5.1 ± 0.7	6.0 ± 0.5	104.6 ± 3.8	5.2 ± 0.1
		20	44.1 ± 3.9	349.6 ± 24.7	161.2 ± 13.0	7.6 ± 0.9	5.7 ± 0.5	145.1 ± 4.2	6.5 ± 0.1
		**Pooled**	**47.7 ± 4.0**	**351.2 ± 27.3**	**116.2 ± 35.8**	**6.0 ± 1.3**	**6.3 ± 0.8**	**110.1 ± 11.8**	**5.6 ± 0.4**
	**Pooled**	**Pooled**	**49.8 ± 5.3**	**362.5 ± 36.6**	**108.4 ± 35.5**	**5.4 ± 1.5**	**6.0 ± 0.7**	**108.3 ± 23.8**	**5.4 ± 0.8**
**Pooled**	**Pooled**	0	51.7 ± 3.9	362.6 ± 32.1	82.6 ± 10.1	4.5 ± 0.7	6.4 ± 0.5	87.9 ± 15.8	4.9 ± 0.6
		10	50.7 ± 5.5	365.4 ± 40.9	91.3 ± 20.6	4.7 ± 1.1	6.2 ± 0.7	93.7 ± 15.3	4.9 ± 0.5
		20	47.2 ± 4.2	347.3 ± 29.0	120.7 ± 33.0	6.0 ± 1.4	6.0 ± 0.6	114.1 ± 15.5	5.5 ± 0.5
**Pooled**	**Pooled**	**Pooled**	**49.8 ± 4.9**	**358.2 ± 35.6**	**98.5 ± 28.4**	**5.1 ± 1.3**	**6.2 ± 0.6**	**98.5 ± 28.4**	**5.1 ± 1.3**
